# RNA Expression Profile and Alternative Splicing Signatures of Genistein-Treated Breeder Hens Revealed by Hepatic Transcriptomic Analysis

**DOI:** 10.1155/2019/3829342

**Published:** 2019-11-25

**Authors:** Zengpeng Lv, Jingle Jiang, Chao Ning, Hongjian Dai, Song Jin, Xihui Wei, Debing Yu, Fangxiong Shi

**Affiliations:** ^1^College of Animal Science and Technology, Nanjing Agricultural University, Nanjing 210095, China; ^2^College of Animal Science and Technology, Shandong Agricultural University, Taian 271018, China; ^3^Changzhou Animal Disease Control Center, Bureau of Agriculture and Rural Affairs of Changzhou, Jiangsu 213003, China

## Abstract

Little information has been available about the influence of dietary genistein (GEN) on hepatic transcriptome of laying broiler breeder (LBB) hens. The study is aimed at broadening the understanding of RNA expression profiles and alternative splicing (AS) signatures of GEN-treated breeder hens and thereby improving laying performance and immune function of hens during the late egg-laying period. 720 LBB hens were randomly allocated into three groups with supplemental dietary GEN doses (0, 40 mg/kg, and 400 mg/kg). Each treatment has 8 replicates of 30 birds. Dietary GEN enhanced the antioxidative capability of livers, along with the increased activities of glutathione peroxidase and catalase. Furthermore, it improved lipid metabolic status and apoptotic process in the liver of hens. 40 mg/kg dietary GEN had the better effects on improving immune function and laying performance. However, transcriptome data indicated that 400 mg/kg dietary GEN did negative regulation of hormone biosynthetic process. Also, it upregulated the expressions of EDA2R and CYR61 by the Cis regulation of neighbouring genes (lncRNA_XLOC_018890 and XLOC_024242), which might activate NF-*κ*B and immune-related signaling pathway. Furthermore, dietary GEN induced AS events in the liver, which also enriched into immune and metabolic process. Therefore, the application of 40 mg/kg GEN in the diet of breeder hens during the late egg-laying period can improve lipid metabolism and immune function. We need to pay attention to the side-effects of high-dose GEN on the immune function.

## 1. Introduction

Genistein (GEN), a type of soy-derived isoflavone, has been demonstrated to be effective in improving metabolism and immune function. Soybean isoflavone exerts either estrogen-like or antiestrogenic effects, which depend on its dose effect [1, 2]. GEN can also activate the PPAR pathway as a ligand, which promotes *β*-oxidation of fatty acid [3]. Recently, our published paper indicated that 40 mg/kg and 400 mg/kg dietary GEN supplementation for LBB hens increased the egg-laying rate during the late laying period, and the adding dose of 40 mg/kg had the better effects [4]. Actually, our previous research suggested that supplementing high-dose (400 mg/kg) and low-dose (40 mg/kg) GEN for laying broiler breeder (LBB) hens made different effects on the immune function of chick embryos [5]. Therefore, we speculate that dietary GEN can alter metabolic process and immune function during the process of liver aging.

The functions of the ovary and fallopian tube of laying hens deteriorate during the late laying stage, which result in rapid decrease in the laying rate [4]. Immune organs, including the thymus and bursa of Fabricius, also degrade in the late laying period. Therefore, the serum antibody titers and immune function of hens decreased in this special stage [6]. Furthermore, caged hens during the late egg-laying period suffer from liver aging along with the increase of hepatic nitric oxide levels, triglyceride, and inflammatory factors, which result in fatty liver syndrome [7, 8]. It raises our attention to the effects of dietary GEN on the metabolism and immune function of LBB hens. Liver aging not only damages the histomorphology but also increases the risk of metabolic abnormalities [9]. GEN is known to act against lipid peroxidation and inflammation [10]. Administration of GEN can reportedly decrease Ovx-induced adiposity and improve insulin sensitivity in retroperitoneal WAT [11, 12]. Our previous data suggest that GEN is effective in ameliorating inflammatory cell infiltration in fatty livers of hens, downregulating TNF-*α*, IL-6, and IL-1*β* expressions [13]. However, a dose-related increase of cytotoxic T cell activity was observed in GEN-treated mice [14]. Recent studies raise the possibility that GEN soy food may be capable of producing thymic and immune abnormalities [15]. To date, the mRNA profile of GEN-treated breeder hens during the late egg-laying period has been unclear.

Long noncoding RNAs (lncRNAs) play important roles in regulating gene expression at epigenetic, transcriptional, and posttranscriptional levels, including genomic imprinting, chromatin modification, and transcriptional activation [16]. Chiyomaru et al. pointed out that GEN can inhibit the reproduction of prostate cancer cells through lncRNA_HOTAIR regulating targeting miR-34a [17]. Our resent research suggested that a novel lncRNA activated by daidzein can stabilize FOS mRNA by serving as a competitive endogenous RNA, which regulated immune function [18]. Therefore, it is of great significance to explore the effect of GEN on lncRNA profiles in the liver of aged LBB hens. Proteins with different functions can be produced by a diverse array of transcripts derived from a single pre-mRNA, suggesting that alternative splicing (AS) is also crucial in regulating gene expression of eukaryotes [19]. 40–60% of human genes have AS isoforms, although some variants exist only in relatively low abundance [20]. We hypothesize that lncRNAs and AS are involved in regulating immune and metabolic processes in older laying hens. In recent years, the study of gene interactions has been broadened considerably because of advances in RNA-Seq, a novel gene expression profiling technology based on high-throughput sequencing [21]. The objectives of this study are to clarify the effects of GEN on the metabolism and immune function of geriatric LBB hens and reveal the RNA profile and alternative splicing signatures by hepatic transcriptomic analysis.

## 2. Materials and Method

### 2.1. Materials

Genistein (GEN) is a synthetic product from Kai Meng. Co. (Xi An, China) Chemical Plant with 99.8% purity.

### 2.2. Animals and Experimental Design

All procedures for animal handling were conducted under protocols approved by the Animal Welfare Committee of Nanjing Agricultural University. Ross 308 LBB hens (57 weeks old) were obtained from the Tushan Breeding Farm (Changzhou, China) and housed under standard conditions. After a 2-week adaptation period, 720 LBB hens with close weight were randomly assigned to 1 of 3 dietary groups. We adjusted the egg production rate of each group to be basically consistent according to the records of laying rate and inverting anus. Each group includes 8 replicates of 30 birds each: a basic diet (CON), CON supplemented with GEN (40 mg/kg diet) (LGE), and CON supplemented with GEN (400 mg/kg diet) (HGE). The formal experiment lasts for 8 weeks. The feed formula is shown in [Supplementary-material supplementary-material-1], which meets the nutritional requirements of LBB hens according to the National Research Council (NRC) guidelines (1994). Each hen was allotted 155 g of feed at 6:00 a.m. every day, with free access to water.

### 2.3. Sample Collection and Chemical Analysis

At 8 weeks of the experiment, one chick each replicate was selected randomly after 10 h of feed deprivation. One blood sample was collected from the wing vein into vacuum tubes (with EDTA) for blood cell detection. Another blood sample was collected for centrifugation (3000×g for 15 min). The serum was stored at –20°C for the measurement of biochemical indices. Then, the hens were killed by decapitation. The liver samples were collected and kept in liquid nitrogen, for detections of RNA-Seq, antioxidative, and metabolic indexes.

### 2.4. Metabolic Indexes in the Serum and Liver

Serum indexes, including glucose (GLU), total glyceride (TG), total cholesterol (TC), and high/low-density lipoprotein cholesterol (HDLC/LDLC), and liver levels of TG, TC, and free fatty acids (FFA) were assayed using assay kits (Unicel DXC 800, Beckman Coulter, California, America).

### 2.5. Serum Levels of Antibody and Immunoglobulin

The serum antibody titers against Newcastle disease (ND) and infectious bursa of Fabricius (IBD) viruses were determined using a commercial ELISA kit (IDEXX Laboratories Inc., Westbrook, Maine, USA) according to the manufacturer's protocol. The serum IgG and IgA levels were determined using a commercial ELISA kit (Bethyl Laboratories Inc., Montgomery, TX, USA) according to the manufacturer's recommended protocol.

### 2.6. Blood Routine Examination

The blood routine examination was performed at Xiyuan Hospital, Beijing, China.

### 2.7. Lymphocyte Classification and Proliferation

The methods and materials of lymphocyte classification and proliferation refer to our previous research [22]. The lymphocytes were mixed with CD3 (SPRD), CD4 (FITC), and CD8 (RPE) antibodies or Bu-1 (RPE) antibodies. The results are expressed as percentages. The proliferative responses of T and B cells were activated by concanavalin A (ConA, 45 *μ*g/mL) and lipopolysaccharide (LPS, 25 *μ*g/mL), respectively, and then were determined by an MTT assay.

### 2.8. Next-Generation Sequencing (NGS)

Total RNA for sequencing was purified from 20 mg of liver samples from twelve chickens (four replicates in each group) using the RNeasy Fibrous Tissue Mini messenger RNA (mRNA) extraction kit (Qiagen, Hilden, Germany). The concentration and purity of total RNA were determined using a UV/Vis spectrophotometer (ACTGene, New Jersey, USA) at 260 nm. RNA integrity was evaluated through a microfluidic assay using a Bioanalyzer system (Agilent Technologies, Inc., Santa Clara, USA). Only high-quality RNA extracts (RNA integrity number (RIN) ≥ 8) were used to pool equal amounts of each RNA sample within a single group. Complementary DNA (cDNA) libraries for RNA sequencing (RNA-Seq) were constructed using a TruSeq RNA Sample Prep Kit v2 (Illumina, San Diego, USA). RNA-Seq analysis was performed to identify transcriptional changes using a MiSeq instrument (Illumina) with paired end libraries (CapitalBio, http://cn.capitalbio.com/). Four replicates from each treatment were analyzed independently for library synthesis and sequencing. The quality of raw reads was assessed using FastQC (Version 0.10.1). Adapters, low-quality reads (*Q* < 20) at the 3′end, reads with fuzzy N bases, ribosomal RNA (rRNA), reads shorter than 20 nt, were trimmed with the FASTX clipper (Version 0.0.13). Paired-end clean reads were mapped to the chicken genome sequence (Gallus_gallus-5.0, version 81, Ensembl) with TopHat v2.0.9 [23]. The gene expression levels in each sample were estimated according to fragments per kilo-base of exon per million fragments mapped (FPKMs) and assessed with Cufflinks v2.1.1 [24]. Transcripts with a *P* < 0.05 were considered differentially expressed.

### 2.9. Prediction and Identification of lncRNA Transcripts

(1) Transcript assembles were conducted using the Cufflinks software. (2) The Cuffmerge software was used to merge all the predicted transcripts. (3) In order to filter out the lncRNA loci encoding proteins, the merged transcripts were mapped to transcripts of known coding proteins downloaded from Ensembl (http://asia.ensembl.org/Gallus_gallus). (4) Size selection and read coverage threshold: only transcripts with ≥200 bp and ≥2 reads were kept. (5) Protein-coding-score test: both the coding-noncoding index (CNCI) and the CPC (coding potential calculator, http://cpc.cbi.pku.edu.cn/programs/run_cpc.jsp) were used to evaluate the coding potential of the candidate lncRNAs [18].

### 2.10. Bioinformatics Analysis

Differential expressed analysis of lncRNA and RNA was carried out using the edgeR software package between LGE *vs*. CON groups and HGE *vs*. CON groups. Low expressed lncRNA and RNA were filtered out before differential analysis. The standard was lncRNA, CPM > 3 in all samples, and CPM > 1 in at least four samples. CPM = *C*/*N*∗1000000, *C* is the read counts that mapped into gene A, and *N* is the total read counts that mapped to all the genes. Further analysis was conducted only using genes that demonstrated a >1.5-fold change or a <0.67-fold change in expression between the groups, as demonstrated by *t*-tests with a *P* value ≤ 0.05. The Database for Annotation, Visualization and Integrated Discovery (http://www.pantherdb.org/) and OmicsBean (http://www.omicsbean.cn) were used to perform gene function enrichment analyses based on gene ontology (GO) and Kyoto Encyclopedia of Genes and Genomes (KEGG) annotation for the significant DEGs.

### 2.11. Identification of Alternative Splicing

The genes of all eukaryotes are discontinuous, with exons interspersed between introns. Alternative splicing (AS) modifies precursor mRNA by removing introns and joining exons, which produce different mRNA transcript units and translate into distinguishable proteins. We used edgeR to analyze the differential expression of transcriptome data at the level of exon and then detect variable shear events by examining the frequency of each gene exon [25].

## 3. Results

### 3.1. Metabolic Indexes

After 8 weeks of GEN treatment, the serum levels of TC and HDLC between the three groups were similar. However, the levels of GLU (*P* < 0.001), TG (*P* = 0.098), and LDLC (*P* = 0.086) in the serum of LGE and HGE groups were lower than those in the CON group (Table 1). The adding levels of 40 mg/kg had better effects on improving the serum indexes. We further detected the metabolic indexes in the liver of LBB hens. As shown in Table 1, dietary GEN supplementation decreased the contents of TC (*P* < 0.01), TG (*P* = 0.078), and FFA (*P* < 0.05) in the liver.

### 3.2. Blood Routine Index and Lymphocyte Classification

The levels of EOS, ALB, and GLB in the serum did not differ significantly between the three groups. Low- and high-dose GEN supplementation significantly increased the blood levels of WBC and BASO (Table 2, *P* < 0.05). The percentages of MONO and NEUT in the peripheral blood of the LGE and HGE groups were numerically higher than those in the CON group (*P* < 0.05). Lymphocyte classification suggested that 40 mg/kg GEN supplementation increased CD3^+^ T lymphocytes and B lymphocytes in the peripheral blood compared to the CON group (*P* < 0.05), while 400 mg/kg GEN supplementation decreased CD4^+^CD8^−^ T lymphocytes and increased CD4^−^CD8^+^ T lymphocytes compared to the CON and LGE groups (*P* < 0.05). In addition, 400 mg/kg dietary GEN decreased CD4^−^CD8^+^ T lymphocytes compared with the LGE groups (*P* < 0.05).

### 3.3. Lymphocyte Proliferation and Serum Antibody and Immunoglobulin Levels

We further detected lymphocyte proliferation in the peripheral blood and serum antibody and immunoglobulin levels to measure the immune level. The serum level of IgA in the LGE and HGE groups was dramatically higher than that in the CON group (Table 3, *P* < 0.05). Similarly, GEN 40 mg/kg treatment increased IBD antibody titer and LPS SI (*P* < 0.05). However, 400 mg/kg GEN decreased LPS SI and IBD antibody titer compared with 40 mg/kg GEN treatment (*P* < 0.05).

### 3.4. Antioxidative Capability in the Liver

Antioxidative capability can reflect the body's redox level. TAOC and GSH-PX activity in the liver of the LGE group was significantly increased compared with that in the CON group (Table 4, *P* < 0.05). 400 mg/kg GEN supplementation for LBB hens numerically enhanced the CAT activity in the liver compared with the CON group (*P* < 0.10). GEN treatment had no effects on MDA levels in the liver.

### 3.5. QC of RNA-Seq Data, Identification of Transcripts, and mRNA Profiles in the Liver

**Figure 1:**
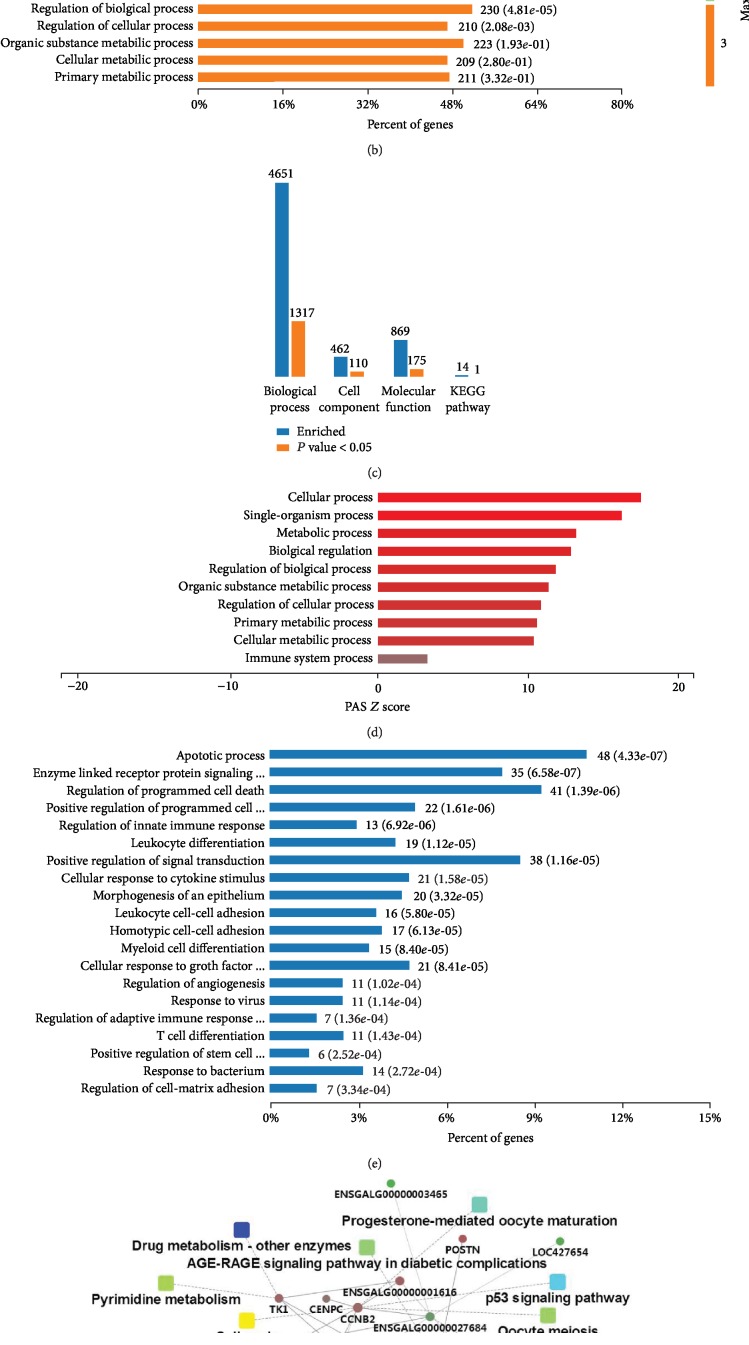
Effects of 40 mg/kg dietary genistein supplementation of LBB hens on differential gene expression and functional enrichment. (a) Number of differentially expressed genes (DEGs) (up/downregulated) in LBB hens after genistein supplementation at 40 mg/kg. (b) Levels 2 and 3 of enriched biological processes. (c) Number of DEGs enriched in GOs and pathways. (d) The top highest PAS *Z* score of enriched biological processes. PAS *Z* score represents the pathway activation strength value, served as the activation profiles of the signaling pathways based on the expression of individual genes. (e) Level 6 of enriched biological processes. (f) Protein-protein interaction analysis using DEGs of the CON *vs*. LGE groups. Circular nodes represent genes/proteins; rectangles represent KEGG pathways or GO biological process terms. The pathways are colored with a gradient from yellow to blue, in which yellow indicates a smaller *P* value and blue indicates a larger *P* value. GO biologic processes are colored red. In the fold change analysis, genes/proteins are colored red for upregulation or green for downregulation.

**Figure 2:**
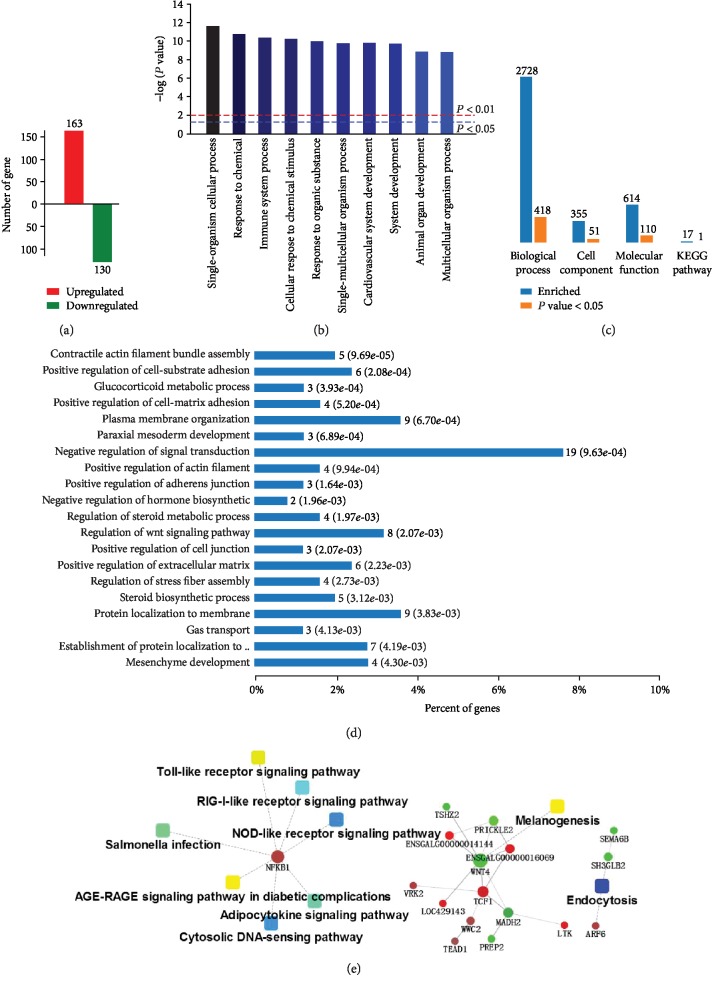
Effects of 400 mg/kg dietary genistein supplementation of LBB hens on differential gene expression and functional enrichment. (a) Number of differentially expressed genes (DEGs) (up/downregulated) in LBB hens after genistein supplementation at 40 mg/kg. (b) Significantly enriched biological processes. (c) Number of DEGs enriched in GOs and pathways. (d) Enriched biological processes of level 6 were shown. (e) Protein-protein interaction analysis using DEGs of the CON vs. LGE groups. Circular nodes represent genes/proteins; rectangles represent KEGG pathways or GO biological process terms. The pathways are colored with a gradient from yellow to blue, in which yellow indicates a smaller *P* value and blue indicates a larger *P* value. GO biological processes are colored red. In the fold change analysis, genes/proteins are colored red for upregulation or green for downregulation.

We established 12 cDNA libraries from the livers of LBB hens in the CON, LGE, and HGE groups, with 4 replicates in each group (Table 5). RNA-Seq generated 78,768,686–94,821,854 raw reads for each library, with an average of 88,374,634, 87,465,006, and 82,427,000 paired-end reads for the CON, LGE, and HGE groups, respectively. Low-quality reads were filtered out, and the average numbers of clean reads were 82,831,718 (93.73%), 81,774,001 (93.49%), and 77,440,227 (93.95%) for the CON, LGE, and HGE groups, respectively. The clean reads were used for all further analyses. After assembly, a total of 17,108 mRNAs was obtained from the 3 groups. An average of 92.45% of the reads in each library was uniquely mapped to the galGal4 assembly of the chicken genome, and the average mapping rates were 92.63%, 92.58%, and 92.15% for the CON, LGE, and HGE groups, respectively (Table 5). We identified 501 DEGs (295 upregulated and 206 downregulated) in the LGE group compared with the CON group (*P* < 0.05) with a |fold change| > 1.5 (Figure 1(a)). The expression abundance and fold changes of DEGs are shown in [Supplementary-material supplementary-material-1]. Meanwhile, we identified 296 DEGs (163 upregulated and 130 downregulated) in the HGE group compared with the CON group (Figure 2(a)).

### 3.6. GO Terms and PPI Analysis

We performed a functional enrichment analysis using the 501 DEGs between the CON and LGE groups, which revealed 1317, 110, 175, and 1 terms associated with biological process regulation, cell components, molecular function, and Kyoto Encyclopedia of Genes and Genomes (KEGG) pathways, respectively (Figure 1(c)). 40 mg/kg GEN treatment mainly affected the gene ontology (GO) terms, including immune system process, single-organism process, biological regulation, cellular process, and metabolic process at the max 2nd and 3rd enriched levels (Figure 1(b)). The PAS *Z* value analysis indicated that 40 mg/kg dietary GEN enhanced the above enriched biological processes (Figure 1(d)). Further cluster analysis suggested that 40 mg/kg dietary GEN affected apoptotic process and did positive regulation of programmed cell death and signal transduction, including enzyme-linked receptor protein signaling pathway at the 6th enriched level (Figure 1(d)). Most genes enriched into the positive regulation of immune system process (GO:0002684), including innate immune response, leukocyte differentiation, cellular response to cytokine stimulus, leukocyte differentiation, myeloid cell differentiation, cellular response to growth factor stimulus, response to virus, regulation of adaptive immune response, T cell differentiation, positive regulation of stem cell differentiation, and response to bacterium (Figure 1(e)). A complete list of the different GO terms identified in this analysis is provided in [Supplementary-material supplementary-material-1]. Cytoscape bioinformatics analysis of potential protein interactions was performed using the software OmicsBean. The DEGs between the LGE group and the CON group were significantly related to the categories P53 signaling pathway, TLR signaling pathway, Gap junction, oocyte meiosis, progesterone-mediated oocyte maturation, AGE-RAGE signaling pathway in diabetic complications, drug metabolism, and cell cycle (Figure 1(f)). A complete list of PPI scores is provided in [Supplementary-material supplementary-material-1].

293 DEGs between the CON and HGE groups were significantly enriched into 418, 51, 110, and 1 terms associated with biological process, cell components, molecular function, and KEGG pathways, respectively (Figure 2(c)). The most significantly enriched terms include single-organism cellular process, response to chemical, immune system process, cellular response to chemical stimulus, response to organism process, cardiovascular system development, system development, system development, animal organ development, and multicellular organismal process at all levels (Figure 2(b)). Further analysis revealed that high-dose GEN affected glucocorticoid metabolism process, steroid biosynthetic process, and Wnt signaling pathway and did negative regulation of hormone biosynthetic process at the 6th enriched level, which is different to the low-dose GEN treatment (Figure 2(d)). Interestingly, PPI analysis showed that 400 mg/kg dietary GEN significantly activates the Toll-like signaling pathway, RIG-1 signaling pathway, NOD-like signaling pathway, Salmonella infection, and AGE-RAGE signaling pathway in diabetic complications. All the signaling pathways were connected by the key gene NF-*κ*B (NFKB1), which were downregulated after high-dose GEN supplementation (Figure 2(d)). A complete list of PPI scores is provided in [Supplementary-material supplementary-material-1].

### 3.7. Overview of lncRNA Profiles

**Figure 3:**
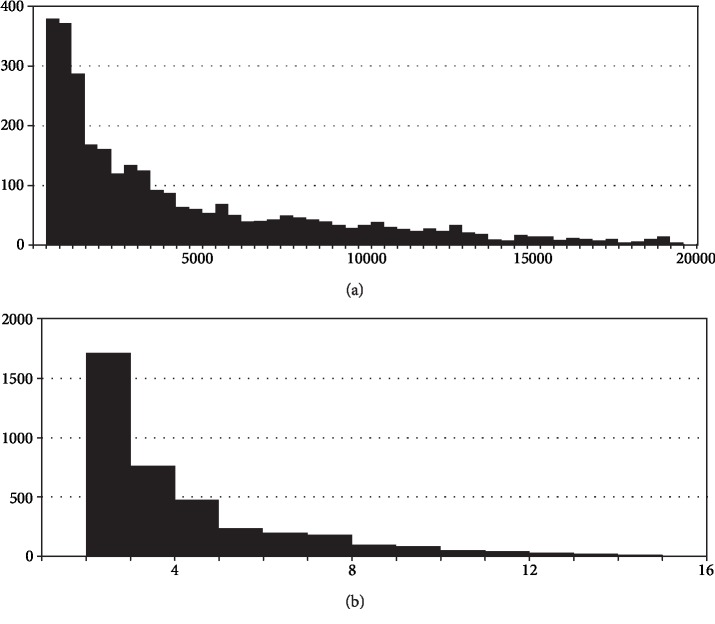
Characteristic analysis of lncRNA transcripts. (a) Distribution of length and frequency of lncRNA transcripts. The *x*-axis represents the number of lncRNAs, the *y*-axis represents the length of lncRNAs (kb). (b) Distribution of exon number of lncRNA transcripts. The *x*-axis represents the number of lncRNAs, the *y*-axis represents the exon number of lncRNA transcripts.

From the lncRNA expression profiles, differentially expressed lncRNAs (DELs) can be found between CON, LGE, and HGE groups. We performed lncRNA screen among the CON, LGE, and HGE groups and identified 1533 lncRNAs ([Supplementary-material supplementary-material-1]). An analysis of all lncRNAs (Figures 3(a) and 3(b)) revealed that the length frequency of lncRNA transcripts was mainly concentrated in 200–5000 units. The number of transcript exons mainly ranged from 2 to 4.

**Figure 4:**
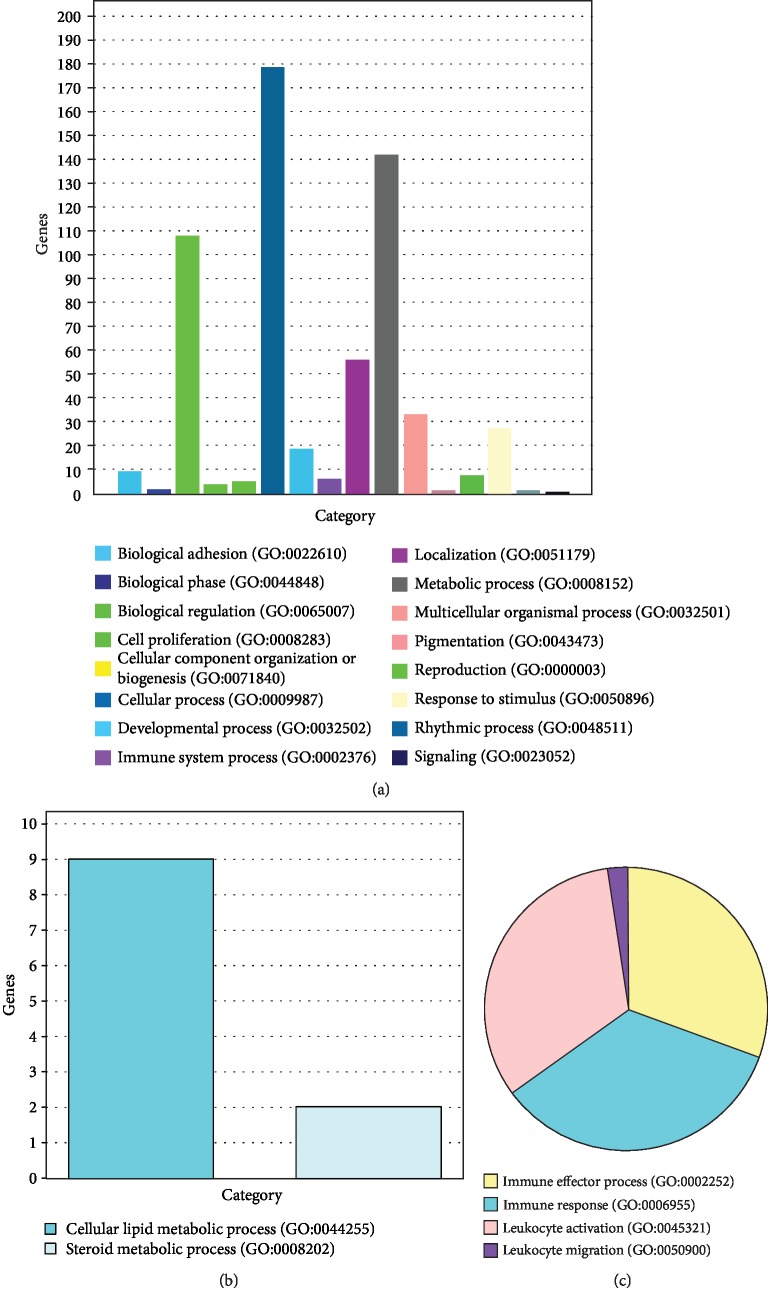
The enriched PANTHER GO-Slim biological processes using lncRNA nearest genes. (a) PANTHER GO-Slim biological processes at all levels. (b) PANTHER GO-Slim biological processes at level 1: immune system process (GO: 0002376). (c) PANTHER GO-Slim biological processes at level 2: primary metabolic process (GO: 0044238).

lncRNA functions mainly by acting on adjacent genes. As we can see from Table 6, there are 1533 intergenic lncRNA loci in the transcriptome of chicken liver. The distances between intergenic lncRNAs and protein-coding genes range from 1 bp to 700 kb. Among them, 45 lncRNAs from scaffold genes (2 overlaps with genes) were annotated, and 1007 lncRNAs were from no scaffold genes (45 overlaps with genes). A total of 438 lncRNAs within 10 kb of the distance to protein-coding genes (including lncRNAs overlapping with genes) were screened out. Panther analysis indicated that the adjacent protein-coding genes of all lncRNAs were significantly enriched into immune system process and metabolic process (Figure 4(a)). As we can see from Figures 4(b) and 4(c), NR2F2 (COUP transcription factor 2), DHCR24 (24-dehydrocholesterol reductase), HNF4A (hepatocyte nuclear factor 4 alpha), SPTLC1 (serine palmitoyltransferase long chain base subunit 1), CYP3A7 (cytochrome P450 family 3 subfamily A polypeptide 7), ECI2 (Enoyl-CoA delta isomerase 2), and PLIN2 (Perilipin) were enriched into cellular lipid metabolic process (GO:0044255) and steroid metabolic process (GO:0008202). DPP10 (dipeptidyl peptidase-like 10), TRAIL (TNF-related apoptosis inducing ligand-like protein), LOC100859408 (class I histocompatibility antigen, f10 alpha chain-related), TLR3 (Toll-like receptor 3), STAT5B (signal transducer and activator of transcription 5B), and LMO4 (LIM domain only 4) were enriched into immune factor process (GO:0002052), immune response (GO:0006599), leukocyte activation (GO:00445321), and leukocyte migration (GO:0050900). Therefore, lncRNAs in the chicken liver are involved in regulating the metabolism and immune function.

Differentially expressed lncRNAs (DELs) were selected from lncRNAs that demonstrated a >1.5-fold change or a <0.67-fold change in expression between the groups, as demonstrated by *t*-tests with a *P* value ≤ 0.05. We identified 70 DELs (34 upregulated and 36 downregulated) in the LGE group compared with the CON group. The expression abundance and fold changes of DEGs are shown in [Supplementary-material supplementary-material-1]. Meanwhile, we identified 66 DEGs (37 upregulated and 29 downregulated) in the HGE group compared with the CON group. We used the adjacent coding genes of DELs to predict their enriched function by Panther signaling pathway analysis. The result indicated that 40 mg/kg dietary GEN enhanced vasopressin synthesis, EGF receptor signaling pathway, interleukin signaling pathway, transcription regulation by b ZIP transcription factor, and FGF signaling pathway. Both 40 and 400 mg/kg dietary GEN significantly enhanced the JAK/STAT signaling pathway (Table 6).

### 3.8. Analysis of Target Genes of lncRNA

Cis regulation mainly depends on Cis-acting elements, including promoters, enhancers, regulatory sequences, and inducible elements, which participate in the regulation of gene expression in the nucleus. Cis-acting elements are usually transcribed into noncoding RNA. If there are DELs and mRNAs in the range of 10 kbp (upstream and downstream), we define that the lncRNAs are involved in the Cis regulation. As shown in Table 7, 40 mg/kg dietary GEN downregulated the expressions of lncRNA_XLOC_010112, as well as the mRNA expressions of their adjacent protein-coding genes (ANKRD33B), respectively. However, 400 mg/kg dietary GEN upregulated the expressions of lncRNA_XLOC_018890 and XLOC_024242, as well as the mRNA expressions of their adjacent protein-coding genes (EDA2R and CYR61), respectively. Therefore, low-dose GEN might Cis downregulate the mRNA expressions of ANKRD33B through lncRNA_XLOC_010112. High-dose GEN might Cis upregulate the mRNA expressions of EDA2R and CYR61 through lncRNA_XLOC_018890 and XLOC_024242, respectively.

### 3.9. Identification of Alternative Splicing (AS)

**Figure 5:**
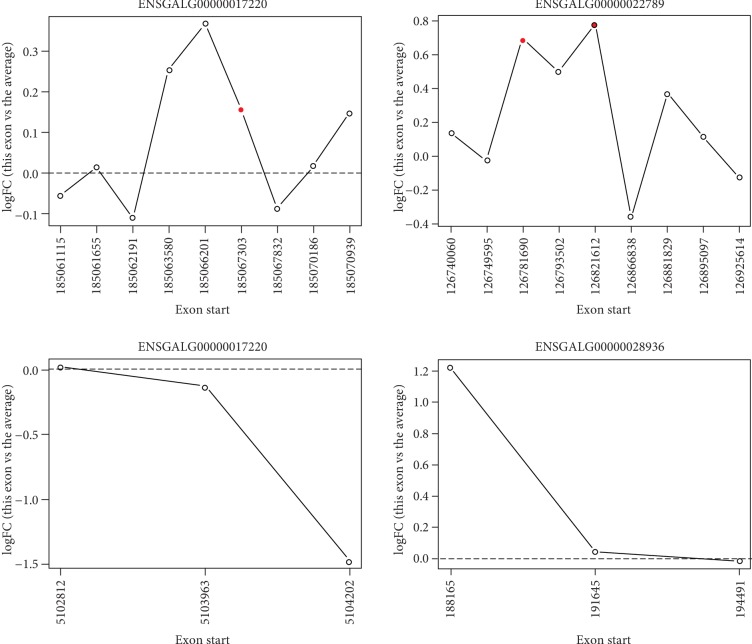
Top four genes with alternative splicing discovered by *F*-test.

**Figure 6:**
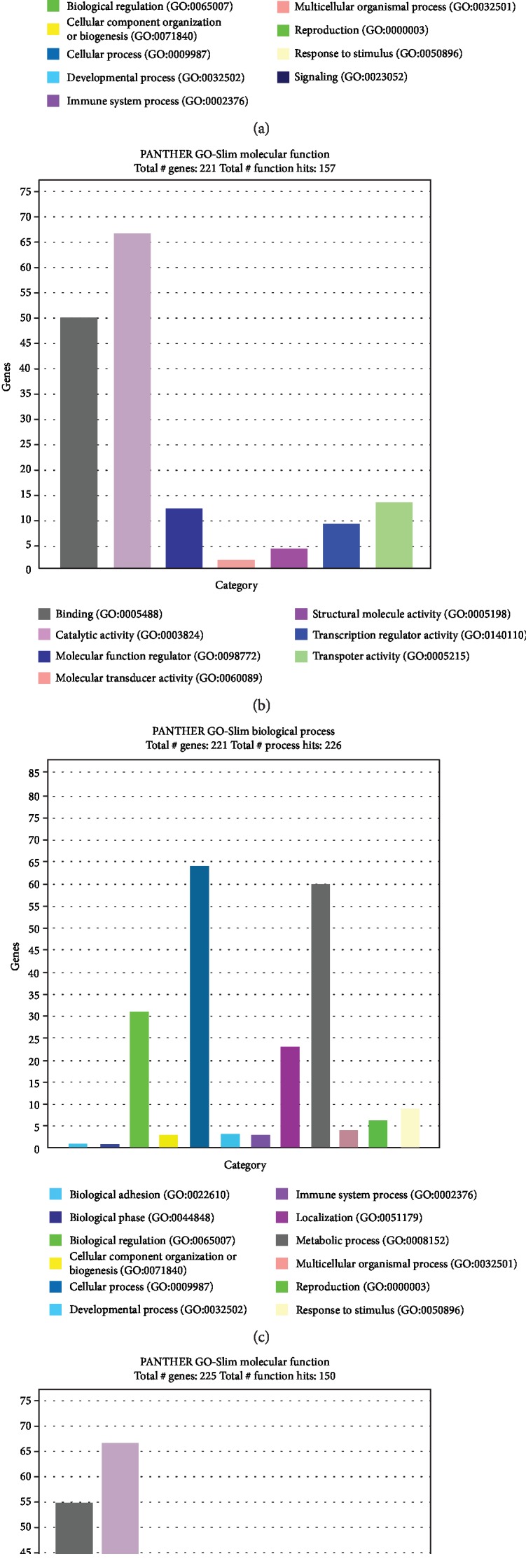
The enriched PANTHER GO-Slim using genes with AS. (a) PANTHER GO-Slim biological processes using genes with AS between LGE and CON groups. (b) PANTHER GO-Slim molecular functions using genes with AS between LGE and CON groups. (c) PANTHER GO-Slim biological processes using genes with AS between HGE and CON groups. (d) PANTHER GO-Slim molecular functions using genes with AS between HGE and CON groups.

AS, a type of gene regulatory process, can generate various mRNA transcript units. It commonly occurs in eukaryotes, which increases the biological diversity of proteins. As shown in [Supplementary-material supplementary-material-1], there were 9894 AS events in the twelve libraries. The top four genes with alternative splicing discovered by *F*-test were shown in Figure 5. The number of AS occurring on the + strands of genes was more than that occurring on the − strands (Table 8). 40 mg/kg dietary GEN induced 312 AS events in the hepatic genes compared with the CON group. Meanwhile, 302 AS events were significantly altered after 400 mg/kg GEN treatment. As we can see from Figure 6, both low- and high-dose dietary GEN influenced immune system, metabolic process, and reproduction through AS events. Furthermore, AS events after GEN treatment influenced molecular functions, such as catalytic activity and transcription regulator activity. Therefore, dietary GEN can alter immune and metabolic processes through AS events.

## 4. Discussion

### 4.1. Dietary GEN Improved the Metabolic Status and Antioxidation

After high-intensity laying cycles, LBB hens during the late egg-laying period suffer from many diseases relating to metabolism and immunity, especially fatty liver syndrome. In the current study, dietary GEN supplementation decreased the contents of TC, TG, and FFA in the liver, as well as the serum levels of TG and LDLC. The result is similar to our previous research that dietary GEN alleviates lipid metabolic disorder in laying hens with fatty liver syndrome [13]. Even so, the effect of dietary GEN on the RNA expression profile of chick livers has not been adequately addressed. Hepatic transcriptome analysis in this study suggested that GEN enhanced the metabolic process in the LBB hens. The adding levels of GEN at 40 and 400 mg/kg both affected the AGE-RAGE signaling pathway in diabetic complications. Therefore, the serum level of glucose was significantly deceased after GEN treatment. AGE can combine with RAGE and activate the MAPK-PI3K pathway, which further induces caspase-3 activation and ROS formation [26, 27]. Accordingly, hepatic transcriptome analysis suggested that low-dose dietary GEN affected the apoptotic process and did positive regulation of programmed cell death and signal transduction. During the process of liver aging, the increasing oxides in mitochondria can do oxidative damages on protein, lipid, and DNA [28]. Excess ROS can further activate mTOR and accelerate liver aging. Antioxidant properties of GEN might be the result of direct chemical actions due mainly to its phenolic structure; it is worth highlighting that GEN can upregulate the expressions of antioxidative phase II enzymes through activation of the Nrf-Keap pathway [29]. In the current study, dietary GEN improved the antioxidative capability of chicken livers, along with the enhanced activity of GSH-PX and CAT. That might be the main reason why dietary GEN can improve the metabolic status and apoptotic process in the liver of LBB hens.

### 4.2. Low-Dose GEN Improved Laying Performance, While High-Dose GEN Inhibited Steroid Biosynthetic Process

GEN and other similar phytoestrogens can exert both estrogenic and antiestrogenic activity, the latter by competing with estradiol for receptor binding [30]. In our previous studies with LBB hens, 40 mg/kg dietary GEN significantly improved the laying performance of laying hens, while 400 mg/kg GEN decreased the laying rate compared with low-dose adding level [4, 13]. The exact mechanisms remain to be elucidated. In the present study, DEGs between the LGE and CON groups were significantly enriched into the biological process, including oocyte meiosis and progesterone-mediated oocyte maturation. Further analysis revealed that high-dose GEN affected glucocorticoid metabolism process, steroid biosynthetic process, and Wnt signaling pathway and did negative regulation of hormone biosynthetic process. Accordingly, our previous research suggested that GEN treatment at 400 mg/kg diet decreased the expression of GnRH in the hypothalamus [13]. Therefore, the dose-dependent effects of GEN on the chick reproduction related to hormone regulation. The current data provides evidences for applying low-dose GEN in the poultry feed.

### 4.3. Low-Dose GEN Improved Immune Function, While High-Dose GEN Might Upregulate NF-*κ*B through Cis Regulation

Liver aging can lead to metabolic disorders, along with inflammatory reactions. The immune system encompasses an array of defenses that help to guard against the development of age-related diseases. However, its functions can be adversely affected by oxidative damage and hormonal changes [31]. What is more, the chicken thymus and bursa of Fabricius degenerate in the late egg-laying period, and the titer of serum antibody drops rapidly. Enhanced immune responses have been found in animals fed with GEN, the isoflavone most abundant in soy food [32, 33]. Isoflavone can reportedly activate PPAR and decrease the expressions of proinflammatory factors (COX-2 and iNOS) [29]. However, the effect of GEN on the immune cells in the peripheral blood is still unknown. Different types of white blood cells participate in the body's defensive response in different ways. Neutrophils can phagocytize bacteria, aging red cells, antigen-antibody complexes, and necrotic cells, playing important roles in innate immunity. Monocytes phagocytize antigens and transfer the antigenic determinants to lymphocytes, which can induce specific immune responses of lymphocytes. In the current experiment, dietary GEN increased the number of leukocytes in the peripheral blood. In particular, the number of monocytes and neutrophils increased after GEN treatment. GEN can reportedly relieve the inflammatory reaction induced by fructose and decrease the level of 8-isoprostaglandin and TNF-*α* in the liver [34]. This leads us to further explore the effect of GEN on acquired immunity of LBB hens. T lymphocyte subsets can fight off viruses through cellular immunity, which depends on the percentages of T lymphocytes (CD3^+^) and its subsets (CD4^+^, CD8^+^) in the peripheral blood [35]. Lymphocyte classification of peripheral blood suggested that 40 mg/kg dietary GEN increased CD3^+^ T and B lymphocytes. In in vitro experiments, GEN shifts the Th1/Th2 balance towards the Th2 response by decreasing the INF*γ*/IL10 ratio [36]. Lipopolysaccharide (LPS) can stimulate B lymphocyte proliferation. In the current experiment, GEN 40 mg/kg significantly increased LPS SI of PBMCs. This indicated that low-dose GEN supplementation improved the humoral immunity of LBB during the late laying stage. Furthermore, we found that GEN 40 mg/kg treatment increased the serum level of IgA and NDV antibody titer. Transcriptomic analysis showed that the DEGs between the LGE group and the CON group were significantly related to the categories P53 signaling pathway and TLR signaling pathway. The most significantly enriched terms include single-organism cellular process, response to chemical, and immune system process. The DEGs and enriched immune signaling pathways were consistent with the immune-phenotypic indices. Therefore, 40 mg/kg GEN could improve the immune function of LBB hens during the late egg-laying period.

Conversely, there is mounting evidence that excess GEN can cause immune dysfunction and decrease cell-mediated immunity [15, 37]. Therefore, we further clarified the effects of high-dose GEN on the immune function of LBB hens. 400 mg/kg GEN supplementation decreased CD4^+^CD8^−^ T lymphocytes and increased CD4^−^CD8^+^ T lymphocytes compared to the LGE group (*P* < 0.05). In addition, 400 mg/kg dietary GEN decreased CD4^−^CD8^+^ T lymphocytes compared with the LGE groups (*P* < 0.05). Compared with CON and 40 mg/kg GEN treatment, 400 mg/kg GEN significantly decreased CD4^+^CD8^−^ T in the peripheral blood and increased cytotoxic T lymphocytes, along with the decreased level of IBD antibody and LPS SI. PPI analysis showed that the enriched immune-related signaling pathways after 400 mg/kg GEN treatment were associated with NF-kappa B. However, 400 mg/kg GEN upregulated the mRNA expression of NFKB1. This is inconsistent with previous research that GEN inhibits the MAPK/NF-*κ*B signaling pathway through tyrosine kinase [38, 39]. Based on the literature and our own observations, we hypothesize that the difference may be related to the dose effect of GEN. lncRNAs play important roles in physiological activities, such as epigenetics, cell cycle, and immune and cell differentiation. Anchor protein repeat ANK (ANKRD33B) can mediate the interaction between proteins, involving cell cycle, transcriptional activity regulation, cell differentiation, and cytoskeleton formation [40, 41]. In this study, transcriptome data predicted that low-dose GEN downregulated the expression of lncRNA and adjacent genes ANKRD33B in the liver. What is more, Collier et al. found that lncRNAs participate in epigenetic modification that relate to inflammation [42]. In the current experiment, transcriptome data predicted that high-dose GEN could upregulate the expressions of EDA2R and CYR61 by the Cis regulation of neighbouring genes (lncRNA_XLOC_018890 and XLOC_024242). Receptor for EDA isoform A2 can activate I-*κ*B kinase/NF-kappaB signaling and JNK cascade. It does positive regulation of NF-*κ*B transcription factor activity. This can explain how high-dose GEN can upregulate the gene expression of NF-*κ*B and activate the inflammation-related signaling pathway. Cysteine-rich protein 61 (Cyr61, CCN1) is an extracellular matrix protein with proinflammatory function, widely involving in pathological damage of inflammation [43, 44]. In this experiment, high-dose GEN might upregulate the expression of CYR61 by Cis regulation of XLOC_024242 and activate the inflammatory signaling pathway. This specific molecular mechanism about Cis regulation needs further verification. Therefore, the lower immune level of HGE-treated hens compared with LGE groups might be due to the Cis regulation of lncRNA.

### 4.4. Dietary GEN Altered AS Relating to Immune Function and Metabolism

AS frequently occurs in eukaryotes. It can generate different mRNA transcript units for a gene, playing important roles in gene expression regulation. In total, we found AS rates with 57.83% of the reference genes in the chick libraries undergoing 3894 AS events. This is much lower than that reported for humans (86.0%) [45], but is higher than that reported for pigs (18.0%) [46] and rice (33.0%) [47]. This phenomenon may result from the differences between eukaryotes in the mechanisms of alternative splicing. In addition, the secondary structure of the precursor mRNA transcript also regulates splicing by bringing splicing elements together [48, 49]. More studies should be conducted to determine gene regulation in the GEN treatment, which shed light on the complexities of lipid metabolism and immune regulation and may provide useful references in addressing issues of affecting liver function and reproductive performance of LBB hens. We further used the genes with AS events to do functional clustering. It indicated that dietary GEN might influence the biological processes, including immune, metabolic process, and reproduction through AS. Meanwhile, the genes with AS were also enriched into molecular functions, including catalytic activity and transcription regulator activity. Although species-specific differences should be considered when comparing chicken with mammalian systems, the current findings appear to be consistent with conservation of lipid metabolism and immune processes in chicken and mammal after GEN treatment. The LBB hen liver transcriptome reported here could greatly broaden our understanding of the regulation and networks of gene expression related to immune and metabolic change after dietary GEN supplementation at different adding levels.

## 5. Conclusion

This study generated transcriptomic data using RNA-Seq technology, which will serve as important resource for revealing the mechanism of immune and metabolic change after GEN treatment. Differences in expressed genes were found between the LGE, HGE, and CON groups, including signaling pathways, splice isoforms, and lncRNAs. Dietary GEN improves the lipid metabolic status of laying hens during the late egg-laying period. 40 mg/kg dietary GEN had the better effects on improving immune function and laying performance, while 400 mg/kg GEN did negative regulation of hormone biosynthetic process and might upregulate NF-*κ*B through Cis regulation of lncRNA. These findings provided scientific basis for the application of phytoestrogens in the animal feed.

## Figures and Tables

**Table 1:** Effects of GEN supplementation on the metabolic indexes of LBB hens during the late egg-laying period.

Indexes	CON	LGE	HGE	*P* value
Serum				
GLU (mmol/L)	10.79 ± 0.38a	8.94 ± 0.35b	9.17 ± 0.28b	<0.001
TG (mmol/L)	17.11 ± 2.15	14.91 ± 2.21	15.11 ± 1.91	0.098
TC (mmol/L)	4.76 ± 0.68	4.15 ± 0.70	4.50 ± 0.75	0.966
HDLC (mmol/L)	0.89 ± 0.22	0.73 ± 0.20	0.72 ± 0.19	0.197
LDLC (mmol/L)	2.77 ± 0.54	2.09 ± 0.64	2.64 ± 0.63	0.086
Liver				
TG (mmol/g, prot)	0.93 ± 0.08a	0.82 ± 0.04b	0.78 ± 0.10b	0.030
TC (mmol/g, prot)	0.96 ± 0.11a	0.87 ± 0.08ab	0.81 ± 0.08b	0.011
FFA (mmol/g, prot)	23.52 ± 3.88	19.73 ± 2.33	22.14 ± 3.65	0.098

GLU: glucose; TG: total glyceride; TC: total cholesterol; HDLC/LDLC: high/low-density lipoprotein cholesterol; FFA: free fatty acids. ^∗^Mean values without a common identifier (a, b) differ significantly between the three groups (*P* < 0.05) (*n* = 8), data expressed as mean ± SD.

**Table 2:** Effects of GEN supplementation on the blood routine indexes of LBB hens during the late egg-laying period.

Treatment	WBC	NEUT	MONO	BASO	EOS	ALB	GLB	CD3^+^ T	CD4^+^CD8^−^ T	CD8^+^CD4^−^ T	B cell
CON	74.98b	16.85b	4.87	0.54b	0.26	22.57	25.29	64.83b	39.84a	9.43b	20.57b
LGE	95.04a	21.29a	5.97	0.76ab	0.21	23.25	28.75	70.72a	43.52a	10.01b	27.26a
HGE	94.15a	20.87a	6.45	0.90a	0.21	23.00	29.75	61.66b	29.03b	14.73a	24.88ab
SEM	3.29	1.09	0.33	0.04	0.02	0.23	0.81	1.35	1.72	2.97	5.14
*P* value	0.029	0.097	0.085	0.015	0.375	0.573	0.217	0.013	<0.001	0.009	0.032

WBC: white blood cell (×10*E*09 cells/L); NEUT: neutrophils (%); MONO: monocytes (%); EOS: eosinophils (%); ALB: albumin (g/L); GLB: globulin (g/L); CD3^+^ T: CD3^+^ T lymphocyte (%); CD4^+^CD8^−^ T: CD4^+^CD8^−^ T lymphocyte (%); CD8^+^CD4^−^ T: CD8^+^CD4^−^ T lymphocyte (%); B cell: B lymphocyte (%); *n* = 8, data expressed as mean and root mean square error (RMSE).

**Table 3:** Effects of GEN supplementation on immune indexes of LBB hens during the late egg-laying period.

Treatment	IBD	NDV	IgG	IgA	LPS SI	ConA SI
CON	3.67b	4.03	11.17	485b	1.26b	1.61
LGE	3.81a	4.04	10.83	605a	1.43a	1.50
HGE	3.65b	4.06	12.34	629a	1.19b	1.60
SEM	0.01	0.01	0.50	21	0.02	0.04
*P* value	0.015	0.074	0.334	0.008	0.009	0.560

LPS SI: lipopolysaccharide stimulation index; ConA SI: concanavalin A stimulation index; IgG: *μ*g/mL; IgM: ng/mL; IgA: *μ*g/mL; *n* = 8, data expressed as mean and root mean square error (RMSE).

**Table 4:** Effects of GEN supplementation on antioxidative indexes of chick livers.

Treatment	TAOC	MDA	CAT	GSH-PX
CON	1.74 ± 0.54b	0.87 ± 0.24	26.45 ± 2.70	49.66 ± 2.50b
LGE	2.19 ± 0.07a	0.93 ± 0.19	28.19 ± 4.09	64.47 ± 4.08a
HGE	1.99 ± 0.13ab	0.86 ± 0.20	31.47 ± 5.45	53.20 ± 5.58b
*P* value	0.037	0.740	0.078	<0.001

TAOC: total antioxidation (U/mg prot); MDA: malondialdehyde (mmol/mg prot); CAT: catalase (U/mg prot); T-SOD: total superoxide dismutase (U/mg prot); GSH-PX: glutathione peroxidase (U/mg prot); data expressed as mean ± SD.

**Table 5:** Characteristics of the reads from 12 chick liver libraries.

Sample ID	Raw reads	*Q*30 (%)	Mean quality score (PF)	Clean reads	Mapped reads 1	Mapped reads 2	Mapping ratio^a^ (%)
CON1	82746656	91.41	35.64	77448096	35948401	35845761	92.70
CON2	92500088	91.42	35.64	86840638	40288053	40218776	92.70%
CON3	93885866	91.34	35.61	87984510	40866183	40787367	92.80%
CON4	84365924	91.44	35.63	79053630	36517429	36434054	92.30%
LEN1	94821854	91.42	35.64	88770278	41256072	41184918	92.90%
LEN2	93846098	91.44	35.64	87990384	40847022	40760507	92.70%
LEN3	82423386	90.54	35.42	76597766	35397144	35297989	92.30%
LEN4	78768686	91.39	35.61	73737576	34108625	34045496	92.40%
HGE1	82198796	91.69	35.68	77223440	35680932	35600982	92.30%
HGE2	81162754	91.77	35.71	76358678	35445783	35371543	92.70%
HGE3	84695016	91.54	35.65	79366240	36085294	36033498	90.90%
HGE4	81651432	91.21	35.59	76812548	35624517	35614277	92.70%

ID: identification; *Q*30: Phred quality score. ^a^Mapping ratio, mapped reads/all reads.

**Table 6:** Distribution of lncRNA and its relationship with genes.

Item	Number
Scaffold	
No gene annotation	481
Gene annotation	45 (2 overlaps with genes)
No scaffold	1007 (45 overlaps with genes)

**Table 7:** Prediction of target genes of lncRNA Cis regulation.

Locus ID	lncRNA_strand	lncRNA_chr	lncRNA_start	lncRNA_end	Gene ID	Gene_chr	Gene_strand	Gene_start	Gene_end	Distance	Gene ID
LGE *vs*. CON											
XLOC_010112	+	2	78212513	78218632	ENSGALG00000027521	2	−1	78170628	78211788	725	ANKRD33B
HGE *vs* CON											
XLOC_018890	−	4	372928	375878	ENSGALG00000004599	4	−1	360637	369568	3360	EDA2R
XLOC_024242	+	8	15139408	15145036	ENSGALG00000008661	8	−1	15135794	15137430	1978	CYR61

**Table 8:** PANTHER classification system statistical overrepresentation test of Cuffdiff results using DEL adjacent genes (LGE *vs*. CON and HGE *vs*. CON).

PANTHER pathways	Gallus gallus (REF)		Client text box input			
	#	#	Expected	Fold enrichment	+/−	*P* value
LGE *vs*. CON						
JAK/STAT signaling pathway	18	1	0.03	36.63	+	0.028
Vasopressin synthesis	25	1	0.04	26.38	+	0.039
EGF receptor signaling pathway	139	4	0.21	18.97	+	<0.001
Interleukin signaling pathway	83	2	0.13	15.89	+	0.007
Transcription regulation by b ZIP transcription factor	56	1	0.08	11.77	+	0.083
FGF signaling pathway	119	2	0.18	11.08	+	0.014
HGE *vs*. CON						
JAK/STAT signaling pathway	18	1	0.02	42.49	+	0.024

## Data Availability

The data of metabolic indexes, blood routine indexes, immune indexes, antioxidative indexes, characteristics of the reads from 12 chick liver libraries, differential gene expression and functional enrichment, distribution of lncRNA and its relationship with genes, characteristic analysis of lncRNA transcripts, PANTHER classification, prediction of target genes of lncRNA Cis regulation, alternative splicing discovered by *F*-test, and PANTHER GO-Slim using genes with AS are included within the article. The data of diet composition, primers, RNA-Seq statistics, differentially expressed genes, complete list of GO terms-biological processes, PPI scores enriched by DEGs, and alternative splicing list used to support the findings of this study are included within the supplementary information files.

## Abbreviations

ALB: Albumin
AS: Alternative splicing
B cell: B lymphocyte
CAT: Catalase
CD3^+^ T: CD3^+^ T lymphocyte
CD4^+^CD8^−^ T: CD4^+^CD8^−^ T lymphocyte
CD8^+^CD4^−^ T: CD8^+^CD4^−^ T lymphocyte
ConA SI: Concanavalin A stimulation index
EOS: Eosinophils
FFA: Free fatty acids
FPKMs: Fragments per kilo-base of exon per million fragments mapped
GEN: Genistein
GLB: Globulin
GLU: Glucose
GSH-PX: Glutathione peroxidase
HDLC/LDLC: High/low-density lipoprotein cholesterol
IBD: Infectious bursa of Fabricius
KEGG: Kyoto Encyclopedia of Genes and Genomes
LBB: Laying broiler breeder
LncRNAs: Long noncoding RNAs
LPS SI: Lipopolysaccharide stimulation index
MONO: Monocytes
ND: Newcastle disease
NEUT: Neutrophils
NRC: National Research Council
RNA-Seq: RNA sequencing
TAOC: Total antioxidative capability
T-SOD: Total superoxide dismutase
TC: Total cholesterol
TG: Total glyceride
WBC: White blood cell.

## Supplementary Materials

Supplementary 1 Table S1: ingredients and analyzed chemical composition of the experimental diets.

Supplementary 2 Table S2: differentially expressed mRNA (LGE vs. CON).

Supplementary 3 Table S3: GO terms-biological processes enriched by DEGs between LGE and CON groups.

Supplementary 4 Table S4: PI scores enriched by DEGs between HGE and CON groups.

Supplementary 5 Table S5: differentially expressed lncRNAs between LGE and CON groups.

Supplementary 6 Table S6: the alternative splicing among the three groups using *F*-test.
