# Supplementary material for: RNA Expression Profile and Alternative Splicing Signatures of Genistein-Treated Breeder Hens Revealed by Hepatic Transcriptomic Analysis
Source: Oxid Med Cell Longev. 2019 Nov 25;2019:3829342. doi: 10.1155/2019/3829342 (PMC6899279; doi:10.1155/2019/3829342)
Supplement: Supplementary 1 — Table S1: ingredients and analyzed chemical composition of the experimental diets. [file 3829342.f1.docx]

**Table S1.** Ingredients, and analyzed chemical composition of the experimental diets

| Ingredient | Content (%) | Nutrient level |  |
| --- | --- | --- | --- |
| Corn | 68.99 | Avian metabolic energy (MC/Kg) | 2.83 |
| Soybean meal | 4.00 | Crude protein (%) | 16.10 |
| Corn protein | 9.15 | Calcium (%) | 3.48 |
| De-gossypol cottonseed protein | 6.00 | Tatalphosphorus (%) | 0.678 |
| Limestone | 7.76 | Available phosphorus (%) | 0.47 |
| Soybean oil | 0.50 | Methionine (% ) | 0.34 |
| Dicalcium phosphate | 2.09 | Lysine (%) | 0.805 |
| NaCl | 0.35 | Met+Cys (%) | 0.626 |
| Trace mineral Premix1 | 0.30 | Threonine (%) | 0.60 |
| Choline chloride (50%) | 0.12 | Tryptophan (%) | 0.18 |
| Mycotoxin adsorbent | 0.10 |  |  |
| DL- Methionine | 0.0515 |  |  |
| Vitamin premix2 | 0.035 |  |  |
| Santoquin | 0.030 |  |  |
| Phytase | 0.016 |  |  |
| 4%Flavomycin | 0.015 |  |  |
| Lysine•HCl(78%) | 0.373 |  |  |
| Threonine | 0.0664 |  |  |
| Tryptophan | 0.0481 |  |  |
| Total | 100.00 |  |  |

^1^Supplied the following per kg complete diet: Cu, 8 mg; Zn, 75 mg; Fe, 80 mg; Mn, 100 mg; Se, 0.15 mg; I, 0.35 mg.
^2^Supplied the following (per kg complete diet): vitamin A, 12500 IU; vitamin D3, 2500 IU; vitamin E, 30 IU; vitamin K3, 2.65 mg; thiamine, 2 mg; riboflavin, 6 mg; vitamin B12, 0.025 mg; biotin, 0.0325 mg; folic acid, 1.25 mg; pantothenic acid, 12 mg; niacin, 50 mg.
